# A Theoretical Study of the Sensing Mechanism of a Schiff-Based Sensor for Fluoride

**DOI:** 10.3390/s22103958

**Published:** 2022-05-23

**Authors:** Sha Ding, Yong Xia, Xiaoqi Lin, Aokui Sun, Xianggang Li, Yuejun Liu

**Affiliations:** 1 Key Laboratory of Advanced Packaging Materials and Technology of Hunan Province, Hunan University of Technology, Zhuzhou 412007, China; 421298314@163.com (S.D.); xiayong@hut.edu.cn (Y.X.); af980808@163.com (X.L.); aksun@hut.edu.cn (A.S.); 2 College of Chemistry and Chemical Engineering, Central South University, Changsha 410083, China

**Keywords:** Schiff-based, TDDFT, TICT, fluoride anion

## Abstract

In the current work, we studied the sensing process of the sensor (E)-2-((quinolin-8ylimino) methyl) phenol (QP) for fluoride anion (F^–^) with a “turn on” fluorescent response by density functional theory (DFT) and time-dependent density functional theory (TDDFT) calculations. The proton transfer process and the twisted intramolecular charge transfer (TICT) process of QP have been explored by using potential energy curves as functions of the distance of N-H and dihedral angle C-N=C-C both in the ground and the excited states. According to the calculated results, the fluorescence quenching mechanism of QP and the fluorescent response for F^–^ have been fully explored. These results indicate that the current calculations completely reproduce the experimental results and provide compelling evidence for the sensing mechanism of QP for F^–^.

## 1. Introduction

Fluoride (F^–^) is an important anion owing to its essential role in industrial, environmental, food, medicinal, and biological science [1]. However, chronic, scarce, and excessive fluoride can lead to a series of adverse effects, such as poor tooth mineralization, saprodontia, severe dental fluorosis, environmental pollution, fluorine poisoning, and even cancer [2,3,4]. Therefore, the recognition and the detection of fluoride anion has attracted a great deal of attention [5,6,7,8].

Compared with other fluoride detection methods, the method of using fluorescence sensors to detect fluoride is more convenient, has high sensitivity, causes no tissue damage, and it is relatively low cost, with good repeatability [9,10,11]. Most of these fluorescence sensors are based on the changes of absorption and emission spectra generated by hydrogen bonding with fluoride anion. The excessive fluoride can break hydrogen bonds, leading to the transfer of proton from the hydrogen bond donor to the acceptor in the excited state. This process generally results in turn-on emission and colorimetric features, defined as excited state intramolecular proton transfer (ESIPT) [12,13,14,15,16]. In contrast, the turn-on fluorescent probe is more popular than the turn-off fluorescent probe, provided the former is more accessible to human eyes. In addition, turn-on fluorescent probes will be more practical in anion recognition with color changes visible to the naked eye. Thus, the ESIPT mechanism is considered to be one of the most efficient and promising methods for designing a fluorescent sensor for fluoride anion. Up to now, the fluoride-binding sites of fluorescent sensors have included pyrrole, amide, thiourea, Shiff base, as well as the hydroxyl unit of salicylaldehyde, which can provide N-H∙∙∙F and O-H∙∙∙F hydrogen bond donors [17,18,19,20,21,22].

**Scheme 1:**
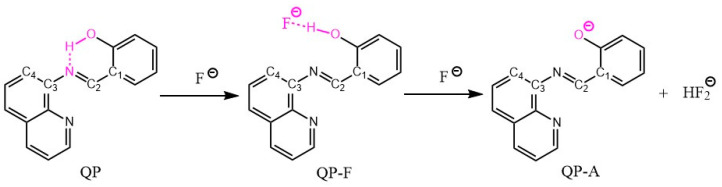
The structure sketches of QP, QP-F and QP-A, numbers only for atomic labeling.

Recently, a novel Schiff-based sensor, which contains a phenol hydroxyl group, has been reported to detect fluoride anion ((E)-2-((quinolin-8ylimino) methyl) phenol (QP), see Scheme 1) [23]. Authors speculated that fluorescence response in the presence of fluoride resulted from the conversion of an intramolecular O-H∙∙∙N hydrogen bond to an intermolecular O-H∙∙∙F hydrogen bond with the occurrence of ESIPT. However, the photophysical properties were not discussed in detail, and the ESIPT process had not been directly proven. Moreover, the other excited state process, twisted intramolecular charge transfer (TICT), caused by the C=N moiety may also occur [24,25]. It is well known that the excited state properties of the fluorescence sensor could be presented conveniently by theoretical calculation [26,27,28,29]. In the current work, the density functional theory (DFT) and the time-dependent density functional theory (TDDFT) calculations are used to clarify the previous fluorescent sensor QP (Scheme 1). The geometries, molecular orbitals, and excitation energies of the sensor QP, fluoride anion complex QP-F, and the deprotonated anion form QP-A are studied both in the ground state and the excited state. The purpose of the current work is to provide a reasonable explanation and in-depth understanding of the fluorescence sensing mechanism of QP for fluoride anion.

## 2. Computational Details

The geometric structures of the ground state (S_0_) and the first singlet excited state (S_1_) were optimized by the DFT and TDDFT methods with PBE0 functional at TZVP basis set [30,31]. There were no symmetry or other constraints during the geometric structure optimization. Then, the vibrational frequency calculations were performed at the same theoretical level, and they confirmed that all of the optimized structures were true local minima. The excitation energies of the lowest six excited states for the QP, QP-F, QP-A, and keto-form of QP (QP-PT) were calculated by the TD-PBE0/TZVP method based on the S_0_ state optimized geometries. The polarized continuum model (PCM) was used to simulate the solvent effects in acetonitrile (MeCN, ε = 35.7) [32]. The calculated absorption and emission energies based on the TD-PBE0/TZVP level are well consistent with the experimental values, which indicate that the current computational level is moderate and suitable for the current system. All DFT and TDDFT calculations were executed within the Gaussian16 program [33].

## 3. Results and Discussion

### 3.1. Geometry Analysis

**Figure 1:**
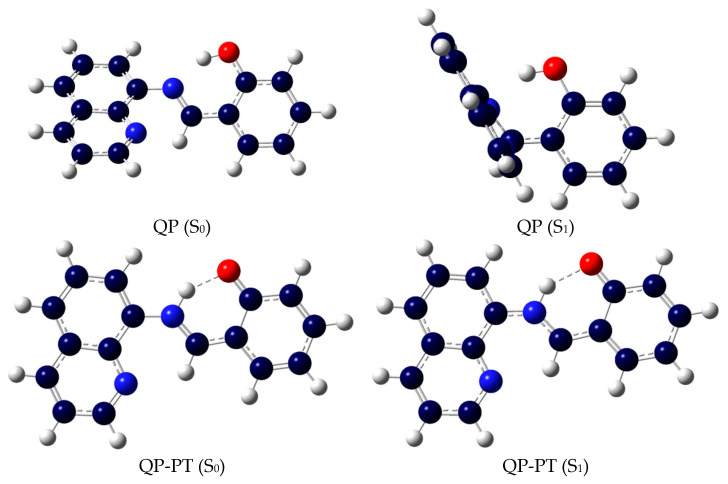
Optimized geometries of QP and QP-PT in the S_0_ and S_1_ states.

**Figure 2:**
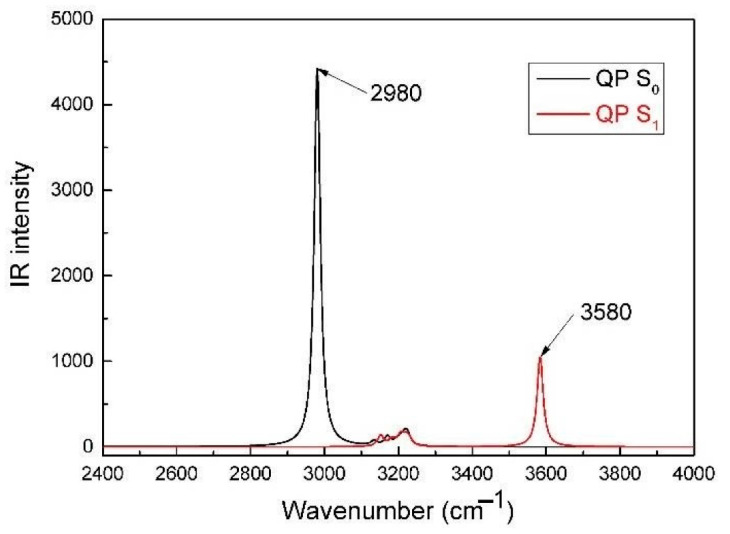
Calculated IR spectra of QP in the S_0_ and S_1_ states.

**Figure 3:**
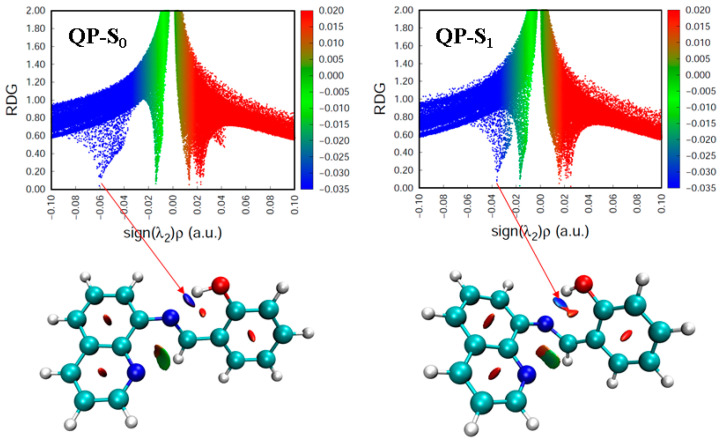
Colored RDG scatter plots (**top**) and isosurfaces (**bottom**) for QP in the S_0_ and S_1_ states. Blue: strong attractive interactions; Green: van der Waals interactions; Red: nonbonding interactions.

The geometries both in the ground (S_0_) and the first excited (S_1_) state are presented in Figure 1 and Figure S1. The selected key geometrical parameters are presented in Table 1 and Table S1. From Table 1, it is clear that when QP is excited from the S_0_ to the S_1_ state, the dihedral angles C_1_–C_2_–N–C_3_ and C_2_–N–C_3_–C_4_ are changed from −178° to −95° and −139° to 175°, respectively. The results indicate the QP molecule possesses lower planarity in the excited state comparing to the ground state. What’s more, the N–H–O bond angle decreases by 6° (4.0%), the N∙∙∙H bond length increases by 0.233 Å (14.1%), and the O-H bond length decreases by 0.03 Å (3.0%) upon excitation. These results indicate the intramolecular hydrogen bonding (O-H∙∙∙N) of QP is weakened with respect to the ground state, which can provide a reliable path for the intermolecular proton transfer process. The infrared (IR) vibrational frequencies of the O-H bond are used to account for the weakness or enhancement of the intermolecular hydrogen bond by means of the electron spectrum blue-shift or red-shift [34,35,36]. The calculated IR spectra of QP in the S_0_ and S_1_ states at the spectral range from 2400 to 4000 cm^−1^ are shown in Figure 2. The calculated O-H bond stretching vibration frequency of QP has a blue-shift of 600 cm^−^^1^ from 2980 cm^−^^1^ in the S_0_ state to 3580 cm^−^^1^ in the S_1_ state, which indicates a weaker hydrogen bond interaction in the S_1_ state. What’s more, the intuitive way in real-space surfaces [37,38,39], the reduced density gradient (RDG) analysis, is adopted to clearly analyze the types and the intensities of intramolecular hydrogen bond (IHB) interactions. Figure 3 presents the colored RDG scatter plots (top) and colored RDG isosurfaces (bottom) for QP in the S_0_ and S_1_ states in which the main concern is the IHB interaction. The contour value of RDG scatter plots sets as 0.1, and the value of RDG isosurface is ranged from −0.035 to 0.02 a.u., in which the blue, green, and red corresponds to hydrogen bonding interactions, van der Waals interactions, and steric crowding effect, respectively. It is obvious that the spike peaks of QP shifts from −0.060 in the S_0_ state to −0.037 in the S_1_ state, indicating a weaker IHB interaction in the S_1_ state, which is consistent with the results of structural analysis and IR spectra. The detail method of RDG analysis is presented in the “Supplementary Materials”. In other words, the O-H of QP is less stabilized, which facilitates the binding of fluorine anion.

The intramolecular O-H∙∙∙N hydrogen bond is broken and a new intermolecular O-H∙∙∙F hydrogen bond is formed after the addition of fluoride. The result is supported by the O-H bond length (1.237 Å) and the F∙∙∙H bond length (1.101 Å) in the ground state QP-F (see Table S1). The addition of excess fluoride anion and the deprotonation of QP may occur at the O-H position. The calculated key geometrical parameters of QP-A shows an insignificant change with respect to the sensor QP. For QP-A, the molecular structures of QP-A in the S_0_ to S_1_ states have almost no change as reflected by the same dihedral angles C_1_–C_2_–N–C_3_ (−177°), which may result in strong fluorescence emission.

### 3.2. Absorption and Frontier Molecular Orbitals

**Figure 4:**
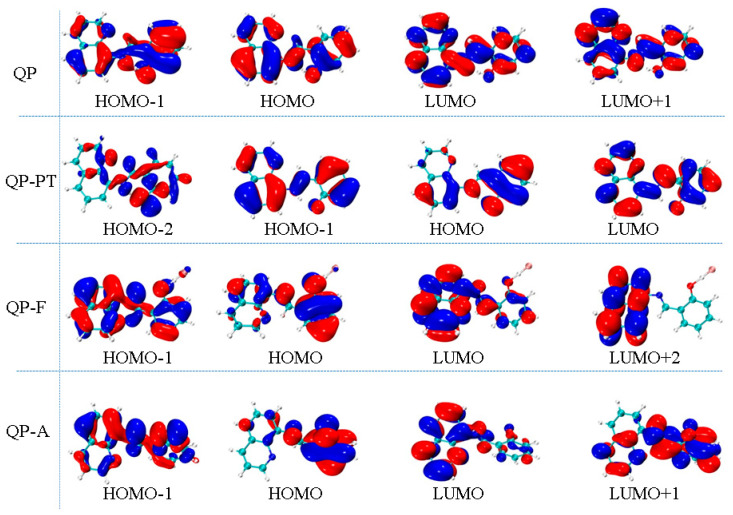
The calculated frontier molecular orbitals for QP, QP-PT, QP-F, and QP-A at TDDFT/PBE0/TZVP level.

As shown in Table 2, the calculated S_0_→S_1_ excitation energy for QP is 352 nm with the oscillator strength *f* = 0.4991, which agree well with the maximum absorption peak at 338 nm in the experiment. The excitation energies of S_0_→S_2_ and S_0_→S_3_ for QP are 315 nm (*f* = 0.0247) and 303 nm (*f* = 0.0043), respectively. The molecular orbitals related to the S_0_→S_1_, S_0_→S_2_ and S_0_→S_3_ transition, which are generated by Multiwfn software [40], are shown in Figure 4. Obviously, the nature of the S_0_→S_1_ transition, which belongs to the highest occupied molecular orbital (HOMO) to the lowest unoccupied molecular orbital (LUMO), is an excitation within the whole molecule, and the S_0_→S_2_ (H–1→L) and S_0_→S_3_ (H→L+1) transition are similar to that of the S_0_→S_1_ transition. Thus, it can be concluded that the transition mode for QP is a local excitation with π→π* type. For QP-F, the calculated S_0_→S_1_ excitation energy is 376 nm with the oscillator strength *f* = 0.3642, which agrees well with the shoulder peak observed in experiment (≈380 nm). It can be seen that the S_0_→S_1_ transition of QP-F, which belongs to the HOMO to the LUMO, has a charge transfer character from the 2-iminomethyl-phenol part to the quinoline unit. For QP-A, the calculated S_0_→S_1_ excitation energy is 432 nm with the oscillator strength *f* = 0.2235, which is very consistent with the maximum absorption peak at 433 nm in the experiment. The nature of the S_0_→S_1_ transition (H→L) of QP-A is similar to that of QP-F, which is a charge transfer from the 2-iminomethyl-phenol part to the quinoline unit.

### 3.3. Proton Transfer and TICT Mechanism

**Figure 5:**
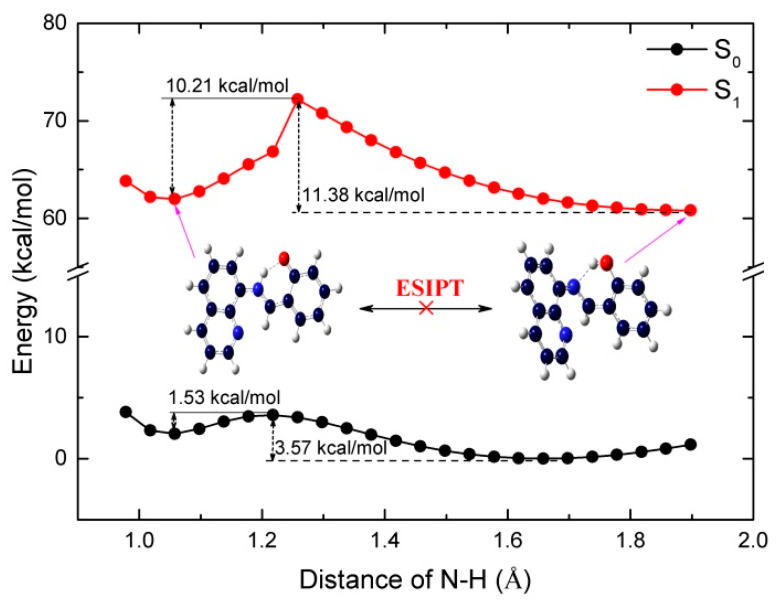
Potential energy curves of QP along with N-H bond length in the S_0_ and S_1_ states.

Figure 5 presents the ground state and the excited state potential energy curves as a function of the N∙∙∙H bond length, which is fixed at values in the range from 0.938 Å to 1.898 Å. As shown in Figure 3, the energy barriers in the ground (S_0_) and the excited (S_1_) states from QP to QP-PT structure are 3.57 and 11.38 kcal/mol, respectively. The results indicate QP can be converted to the QP-PT structure in the S_0_ state, while the energy barrier is too high to overcome in the excited state. Meanwhile, the energy barriers of the reverse process that from QP-PT to QP in the S_0_ and S_1_ states are 1.53 and 10.21 kcal/mol, respectively. The energy barrier in the S_0_ state, which is very low and easy to overcome, is even lower than that of QP to QP-PT. In other words, the two isomers of QP and QP-PT may coexist in the ground state. The lowest energy position in the S_0_ state appears at the length of the O-H bond and it is 1.005 Å, while the N-H bond is 1.658 Å, which corresponds to the optimized ground state geometry of QP. These results indicate the proton transfer of the sensor QP can occur in the ground state, while the excited state intramolecular proton transfer (ESIPT) process does not happen very easily.

**Figure 6:**
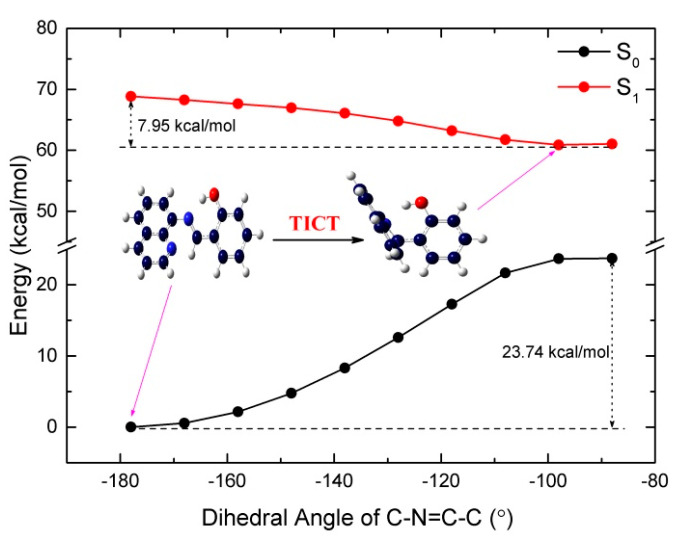
Potential energy curves of QP along with the dihedral angle of C-N=C-C in the S_0_ and S_1_ states.

Currently, the C=N isomerization process, a nonradiative decay path of the excited state, is widely used to design a turn-on fluorescent probe [41,42,43]. In order to elucidate whether the weak fluorescence of QP is derived from the C=N isomerization process, the potential energy curves of QP as a function of the dihedral angle C-N=C-C fixed at values in the range from −178° to −88° were constructed both in S_0_ and S_1_ states. As shown in Figure 6, the energy barrier of the C=N isomerization process for QP in the S_0_ state is monotonically increasing. The energy of the C=N isomerization product is 23.74 kcal/mol higher than that of QP. That is to say, QP is existing in the form of QP rather than a C=N isomerization product of QP in the ground state. Notably, the energy barrier of the C=N isomerization process for QP is almost zero in the S_1_ state. The result indicates that the twisting process of QP in the S_1_ state is spontaneous, reducing the energy gap between the ground state and the excited state. The lowest energy position corresponds to the optimized S_1_ state geometry of QP. The spontaneous emission to the S_0_ state with a negligibly small oscillator strength (*f* = 0.0033, Table 2) suggests a nonradiative transition character. Therefore, the TICT process for QP in the excited state is easy to proceed, which agrees well with the experimental results, and it provides a possible explanation for the weak fluorescence of QP.

### 3.4. Fluorescence Mechanism

**Figure 7:**
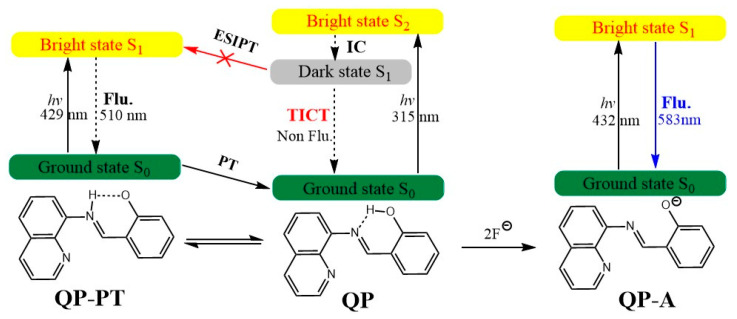
The entire fluorescence sensing processes for the chemosensor QP.

In the S_1_ state, QP is transformed into the C=N isomerization product via the TICT process with the quinolin-8-ylimino group. However, the S_1_ state of the isomerization product is a dark state because of the negligibly small oscillator strength of the S_1_→S_0_ transition. Thus, the excited state decays to the ground by a nonradiative pathway, and the fluorescence of QP is very weak. That is to say, the fluorescence of QP is quenched owing to the TICT process in the excited state rather than the ESIPT process, which is different from the reported mechanism [23,44]. This could be supported by the relevant literature reports, in which the structure of the fluorophore in the literatures are similar to QP [25]. In addition, the calculated results are in good agreement with the experimental phenomenon with the weak fluorescence of QP [23]. After the excessive addition of fluoride, the deprotonation of QP may occur at the O-H position. We obtained the stable structures of QP-A both in the S_0_ and S_1_ states. The calculated fluorescence maximum of QP-A in the S_1_ state is located at 583 nm with *f* = 0.4619, which is obviously red-shifted compared with the experimental result. In order to test the functional effect of the absorption and the emission spectra of QP-A, the range separated hybrid functionals HSE06 [45,46] and lc-BLYP [47] were selected to simulate ultraviolet absorption and the fluorescence band of QP-A according to the previous method [48], and the calculated results are shown in Table S2. The calculated results indicate that the S_1_ state of QP-A is a bright state (*f* = 0.2930 at HSE06/TZVP level, *f* = 0.6131 at lc-BLYP/TZVP level), and the predicted fluorescence maxima of QP-A (407 nm) in the S_1_ state agrees well with the experimental result (389 nm). According to the above results, the sensing mechanism of QP for fluoride can be depicted in Figure 7. The S_1_-state of the receptor QP decays to the ground via a TICT process rather than an ESIPT, providing a pathway for nonradiative transition, which is the reason for the weak fluorescence. The excess addition of fluoride anion results in the deprotonation of the O–H bond, which is driven by the HF_2_‾ complex. The S_1_-state geometry for the deprotonated anion form QP-A is similar to that of S_0_-state with a charge transfer excited feature, which is responsible for the strong fluorescence. Therefore, QP can be used as an excellent candidate for a “turn on” type fluorescent sensor for fluoride anion.

## 4. Conclusions

The new insight into the fluorescent sensing mechanism of QP for fluoride anion is deeply described in the current work by using DFT/TDDFT methods. The optimized geometries and the potential energy curves indicated that the ESIPT process of QP is unlikely to proceed in the excited state with up to 11.38 kcal/mol energy barriers. While the TICT process is spontaneous with a barrierless potential energy curve in the S_1_ state, which is reasonably explained for the weak fluorescence of QP. According to the optimized geometries, the molecular orbitals and the transition information of QP-A, the S_1_-state is a charge transfer excited state with π→π* transition feature, which is responsible for the strong fluorescence. Our work provides a detailed fluorescence quenching mechanism of QP, which is expected to be applied in designing more available fluorescent sensors.

## Figures and Tables

**Table 1:** Calculated key geometrical parameters for QP and QP-PT by the DFT/TDDFT methods.

	QP		QP-PT	
	S_0_ State	S_1_ State	S_0_ State	S_1_ State
Bond length (Å)	data	data		
O–H	1.004	0.974	1.638	1.742
N–H	1.658	1.891	1.050	1.037
Bond angle (˚)				
N–H–O	149	143	144	142
Dihedral angle (˚)				
C_1_–C_2_–N–C_3_	−178	−95	180	180
C_2_–N–C_3_–C_4_	−139	175	−177	−180

**Table 2:** The calculated electronic transition information for QP, QP-PT, QP-F, and QP-A in acetonitrile at TDDFT/PBE0/TZVP level, and corresponding experimental values.

	Transition	Energy nm (eV)	*f*	Composition	CI [%]
QP	S_0_→S_1_	352 (3.52)	0.4991	H→L	95
	S_0_→S_2_	315 (3.93)	0.0247	H–1→L	84
	S_0_→S_3_	303 (4.09)	0.0043	H→L+1	84
	S_1_→S_0_	1167 (1.06)	0.0033	L→H	98
QP-PT	S_0_→S_1_	429 (2.89)	0.4435	H→L	99
	S_0_→S_2_	358 (3.46)	0.0007	H–2→L	91
	S_0_→S_3_	329 (3.76)	0.3898	H–1→L	97
	S_1_→S_0_	510 (2.43)	0.5508	L→H	99
QP-F	S_0_→S_1_	376 (3.30)	0.3642	H→L	93
	S_0_→S_2_	338 (3.67)	0.0505	H–1→L	73
	S_0_→S_3_	324 (3.82)	0.0508	H→L+2	73
	S_1_→S_0_	1030 (1.20)	0.0010	L→H	98
QP-A	S_0_→S_1_	432 (2.87)	0.2235	H→L	98
	S_0_→S_2_	370 (3.34)	0.1045	H–1→L	74
	S_0_→S_3_	352 (3.52)	0.1756	H→L+1	89
	S_1_→S_0_	583(2.12)	0.4619	L→H	97

## Data Availability

Not applicable.

## Supplementary Materials

The following supporting information can be downloaded at: https://www.mdpi.com/article/10.3390/s22103958/s1, Figure S1: Optimized geometries of QP-Fand QP-A in the S_0_ and S_1_ states; Table S1: Calculated key geometrical parameters for QP-F and QP-A by the DFT/TDDFT methods; Table S2: Calculated electronic transition energy for QP-A in acetonitrile at lc-BLYP/TZVP, PBE0/TZVP, HSE06/TZVP levels, and corresponding experimental values.
