# Supplementary material for: A Theoretical Study of the Sensing Mechanism of a Schiff-Based Sensor for Fluoride
Source: Sensors (Basel). 2022 May 23;22(10):3958. doi: 10.3390/s22103958 (PMC9144756; doi:10.3390/s22103958)
Supplement: Supplementary file 1 [file sensors-22-03958-s001.zip › sensors-1688900-supplementary.pdf]

Supporting Information

A Theoretical Study of the Sensing Mechanism of a Schiff-Based Sensor for Fluoride

Sha Ding [1], Yong Xia [1,2], Xiaoqi Lin [1], Aokui Sun [1], Xianggang Li [1,*] and Yuejun Liu [1,*]

[1] Key Laboratory of Advanced Packaging Materials and Technology of Hunan Province, Hunan University of Technology, Zhuzhou 412007, China; 421298314@163.com (S.D.); xiayong@hut.edu.cn (Y.X.); af980808@163.com (X.L.); aksun@hut.edu.cn (A.S.)

[2] College of Chemistry and Chemical Engineering, Central South University, Changsha 410083, China

* Correspondence: lixianggang@hut.edu.cn (X.L.); yjliu_2005@126.com (Y.L.)

Table S1. Calculated key geometrical parameters for QP-F and QP-A by the DFT/TDDFT methods.

	QP-F		QP-A	
	S_0 state	S_1 state	S_0 state	S_1 state
Bond length (Å)				
O–H	1.237	1.172		
F–H	1.101	1.161		
Bond angle (°)				
O–H–F	177	177		
Dihedral angle (°)				
C_1–C_2–N–C_3	-177	-101	-177	-177

Table S2. Calculated electronic transition energy for QP-A in acetonitrile at lc-BLYP/TZVP, PBE0/TZVP, HSE06/TZVP levels, and corresponding experimental values.

	Transition	Energy nm(eV)	*f*
lc-BLYP/TZVP	$S_1 \rightarrow S_0$	407 (3.05)	0.6131
PBE0/TZVP	$S_1 \rightarrow S_0$	583 (2.12)	0.4619
HSE06/TZVP	$S_1 \rightarrow S_0$	582 (2.13)	0.2930
Experiment		389	
lc-BLYP/TZVP	$S_0 \rightarrow S_1$	338 (3.66)	0.6496
PBE0/TZVP	$S_0 \rightarrow S_1$	432 (2.87)	0.2235
HSE06/TZVP	$S_0 \rightarrow S_1$	456 (2.72)	0.1575
Experiment		433	

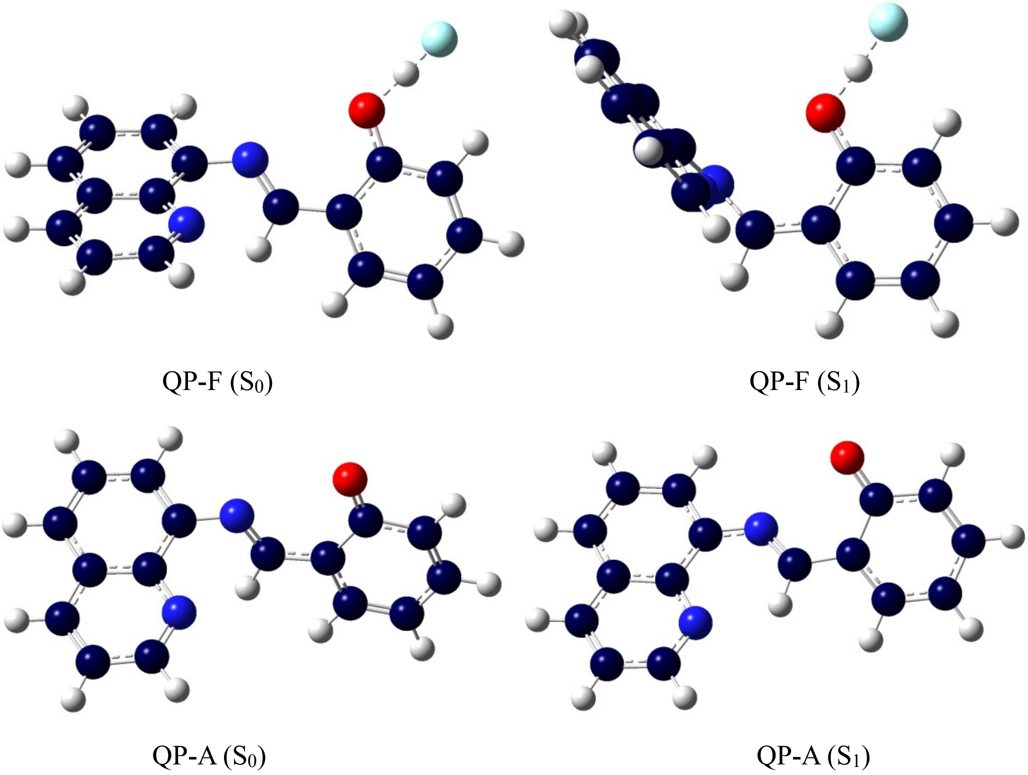

Figure S1. Optimized geometries of QP-Fand QP-A in the S_0 and S_1 states.

Intramolecular hydrogen bond (IHB) interaction

A visual method in real-space surfaces [1], plots of the reduced density gradient (RDG) versus the electron density (ρ) multiplied by the sign of the second Hessian eigenvalue (λ_2), is adopted to clearly analyze the types and intensities of IHB interactions. The y-coordinate RDG and the x-coordinate Ω are expressed as Equation (1) and Equation (2), respectively.

$$RDG = \frac{1}{2(3\pi^2)^{1/3}}\frac{|\nabla\rho|}{\rho^{4/3}} \quad (1)$$

$$\Omega = \mathrm{sign}(\lambda_2)\rho \quad (2)$$

According to Bader's atoms-in-molecules (AIM) theory [2], the sign of the second Hessian eigenvalue $\lambda_2 < 0$ and $\lambda_2 > 0$ represent bonded and non-bonded interactions, respectively. Thus, combined with the electron density ρ, we can qualitatively determine the types and intensities of the interactions. Large, negative values of sign(λ_2)ρ are indicated attractive interactions (such as hydrogen bonding or dipole-dipole); while if the values are large and positive, the interactions are steric crowding. Values neighboring zero represent very weak, van der Waals interactions. The colored RDG scatter plots are constructed by Mulitiwfn [3], and colored RDG isosurfaces are obtained by VMD software [4].

[1] Johnson, E.R.; Keinan, S.; Mori-Sanchez, P.; Contreras-Garcia, J.; Cohen, A.J.; Yang, W. Revealing noncovalent interactions. *J. Am. Chem. Soc.* **2010**, *132*, 6498–6506.

[2] Bader, R.F.W. A quantum theory of molecular structure and its applications. *Chem. Rev*. **1991**, *91*, 893–928.

[3] Lu, T.; Chen, F. Multiwfn: A multifunctional wavefunction analyzer. *J. Comput. Chem.* **2012**, *33*, 580–592.

[4] Humphrey, W.; Dalke, A.; Schulten, K. VMD: Visual molecular dynamics. *J. Mol. Graph. Model*. **1996**, *14*, 33–38.
